# Caffeine Consumption and Heart Rate and Blood Pressure Response to Regadenoson

**DOI:** 10.1371/journal.pone.0130487

**Published:** 2015-06-22

**Authors:** Abbas Bitar, Ronald Mastouri, Rolf P. Kreutz

**Affiliations:** Krannert Institute of Cardiology, Indiana University School of Medicine, Indianapolis, Indiana, USA; University of Bologna, ITALY

## Abstract

**Background:**

Current guidelines recommend that caffeinated products should be avoided for at least 12 hours prior to regadenoson administration. We intended to examine the effect of caffeine consumption and of timing of last dose on hemodynamic effects after regadenoson administration for cardiac stress testing.

**Methods:**

332 subjects undergoing regadenoson stress testing were enrolled. Baseline characteristics, habits of coffee/caffeine exposure, baseline vital signs and change in heart rate, blood pressure, percent of maximal predicted heart rate, and percent change in heart rate were prospectively collected.

**Results:**

Non-coffee drinkers (group 1) (73 subjects) and subjects who last drank coffee >24 hours (group 3) (139 subjects) prior to regadenoson did not demonstrate any difference in systolic blood pressure, heart rate change, maximal predicted heart rate and percent change in heart rate. Systolic blood pressure change (15.2±17.1 vs. 7.2±10.2 mmHg, p = 0.001), heart rate change (32.2±14 vs. 27.3±9.6 bpm, p = 0.038) and maximal predicted heart rate (65.5±15.6 vs. 60.7±8.6%, p = 0.038) were significantly higher in non-coffee drinkers (group 1) compared to those who drank coffee 12–24 hours prior (group 2) (108 subjects). Subjects who drank coffee >24 hours prior (group 3) exhibited higher systolic blood pressure change (13±15.8 vs. 7±10.2, p = 0.007), and heart rate change (32.1±15.3 vs. 27.3±9.6, p = 0.017) as compared to those who drank coffee 12–24 hours prior to testing (group 2).

**Conclusions:**

Caffeine exposure 12–24 hours prior to regadenoson administration attenuates the vasoactive effects of regadenoson, as evidenced by a blunted rise in heart rate and systolic blood pressure. These results suggest that caffeine exposure within 24 hours may reduce the effects of regadenoson administered for vasodilatory cardiac stress testing.

## Introduction

Myocardial perfusion imaging (MPI) has been extensively used for detecting coronary artery disease, assessing viable myocardium, and evaluating the effect of different therapeutic interventions [1]. Vasodilator stress testing accounts for up to 45% of MPI studies [2]. Adenosine and dipyridamole have been the main vasodilators used for stress testing until 2008 when regadenoson was approved by the FDA [3]. Regadenoson is a selective adenosine A2A receptor agonist. It has a very low affinity for A1 adenosine receptors and almost no affinity for A2B and A3 adenosine receptors. Regadenoson’s selectivity for A2A receptors results in increased vasodilation and subsequently increased coronary blood flow (CBF). It is administered at a fixed dose of 0.4mg as an intravenous bolus. It has a rapid onset of action and a short duration of action with maximal plasma concentration achieved within 1 to 4 minutes after administration and an initial half-life of 2 to 4 minutes. [4–6].

The American Society of Nuclear cardiology recommends against the consumption of caffeinated food andbeverages or foods containing other methylxanthines (chocolate) for at least 12 hours prior to regadenoson administration (ASNC 2009). Caffeine, a (1,3,7-trimethylxanthine), is a non-selective competitive inhibitor of all adenosine receptors particularly A2A receptors [7,8].

Few studies assessed the interaction of regadenoson and caffeine. In conscious dogs, intravenous administration of caffeine at 1–10 mg/kg, followed by regadenoson injection 45 minutes later, did not significantly affect regadenoson induced coronary blood flow (CBF), but reduced the duration of the 2-fold increase in CBF. It also blunted heart rate and blood pressure change [9]. In a randomized, placebo-controlled, double blind and crossover pilot study of healthy subjects, Gaemperli et al. showed that moderate caffeine intake 2 hours prior to regadenoson administration did not affect myocardial blood flow (MBF) and blood pressure response but resulted in a blunted heart rate response [10]. In contrast another randomized placebo controlled study demonstrated that administration of 200 or 400 mg of caffeine 90 minutes before regadenoson significantly reduced the number of segments with reversible defects by MPI [11].

The effect and duration of caffeine exposure on blood pressure, heart rate, and percent of maximal predicted heart rate among subjects undergoing pharmacological stress testing with regadenoson is not clear. We hypothesized that chronic caffeine intake with only 12–24 hours cessation prior to regadenoson stress testing according to drug labeling recommendation, affects maximal heart rate and blood pressure response as compared to non-caffeine consumption or more prolonged interruption. The aim of the current study is to assess the effect of habitual caffeine consumption on blood pressure, heart rate, percentage of predicted heart rate and percentage change in heart rate among subjects undergoing vasodilator stress testing with regadenoson.

## Methods

### Patients

The study protocol was approved by the Indiana University institutional review board for research. Written informed consent was obtained from all subjects. Subjects referred for regadenoson stress testing were enrolled. Patients with combined exercise and regadenoson stress testing were excluded from analysis. As per the protocol of our institution, all patients were asked to not consume caffeinated beverages or xanthine containing foods for at least 12 hours prior to study. Moreover, all patients were asked to take their routine daily medications. Baseline demographic data and medical comorbidities were collected on all subjects. Information on amount, frequency, and last exposure to caffeine, chocolate, and caffeinated soft drinks were collected prospectively prior to performance of cardiac stress test. Caffeine exposure was classified according to none recently, last exposure of at least one cup of coffee 12–24 hours prior to regadenoson stress test, and >24 hours prior to stress test. Consumption of one cup of black or green tea was considered equal to one cup of coffee, and subjects who consumed tea were included in the coffee consumption group for analysis. Subjects’ heart rate (HR), and blood pressure were recorded at baseline instantaneously before administration of regadenoson. Subjects remained in a supine position throughout the test. Change in heart rate (changeHR) during the stress test was calculated by subtracting resting (restingHR) from peak heart rate (peakHR) recorded within 5 minutes after administration of regadenoson. Change in systolic blood pressure (changeSBP) was calculated by subtracting resting (restingSBP) from peak systolic blood pressure (peakSBP) recorded within 5 minutes after administration of regadenoson. Maximal predicted heart (MHR) rate was calculated using 220-age (years) and percent maximal predicted heart rate (%MPHR) was calculated by peakHR over MHR and multiplying by 100 (%MPHR = (peakHR/MHR)*100). Percent change in heart rate (%ChangeHR) was calculated by changeHR over restingHR and multiplying by 100. Incidence of patient reported side effects were prospectively recorded (dyspnea, nausea, flushing, dizziness, abdominal pain, headache, chest pain).

Non-coffee drinkers (group 1) were compared to subjects who had consumed coffee within 12 to 24 hours (group 2) or more than 24 hours (group 3) prior to regadenoson administration.

### Statistical Analysis

Baseline demographic and clinical variables are descriptively summarized. Continuous variables are expressed as mean ± SD. Categorical data are presented as percent frequency. Unpaired two-sided Student’s t-test was used to compare normally distributed continuous data between two groups. One-way analysis of variance test (ANOVA) and post hoc Tukey comparisons were used to determine difference between different groups based on coffee consumption. Categorical variables were compared using the χ^2^ test and continuous variables were computed using student t test. Statistical significance was defined as p-value < 0.05.

Multivariable linear regression with change in systolic blood pressure (SBP), HR, and percent maximal predicted heart rate achieved at peak exercise as outcome variables was performed for non-coffee drinkers, subjects exposed to coffee 12 to 24 hours and more than 24 hours before regadenoson administration. Exposure to coffee 12 to 24 hours prior was used as the reference category. Adjustment for known confounders was based on clinical variables known to affect caffeine metabolism, as well as clinical variables with *p*<0.1 in univariable analysis and if adjustment for the variables resulted in at least a 10% change in the estimate of the overall association.

## Results

Baseline characteristics of the study subjects are described in (Table 1). Subjects mean age was 60±11 years. Among the subjects, 257 (78%) were coffee drinkers while 73 subjects denied any coffee consumption. Non-coffee drinkers tended to be younger (57.2±10.5 vs. 60.9±10.5 years, p = 0.01), more obese (105.7±30.7 vs.97.3±23.5 Kg., p = 0.014), consumed less chocolate (69.9% vs. 80.9%, p = 0.042), had more GERD (41.1% vs. 28.4%, p = 0.039) and were less frequently prescribed antiplatelet medication (37% vs. 52.9%, p = 0.016) as compared to coffee consumers. Twelve coffee drinkers did not report when they last consumed coffee. None of the subjects was taking theophylline. Table 1 summarizes baseline characteristics between non-coffee drinkers (group 1), subjects who drank coffee 12–24 hours prior (group 2) and those who drank coffee more than 24 hours to stress testing (group 3).

**Table 1:** Patient Characteristics.

	Total	Non coffee drinkers	12–24 hours hold	>24 hours hold	P Value^1^	P Value^2^	P Value^3^	P Value^4^
Patients	332	73	108	139				
Age (years)	60±10.6	57.2±10.5	62±9.9	59.8±10.6	**0.007**	0.208	0.215	**0.01**
Male (%)	145 (43.7)	35 (47.9)	48 (44.4)	57 (41)	0.643	0.333	0.588	0.616
White (%)	232 (69.6)	48 (65.8)	88 (81.5)	89 (64)	**0.018**	0.803	0.003	**0.007**
Weight (kg)	99.2±25.4	105.7±30.7	95.9±20.6	98.5±25.7	**0.033**	0.132	0.710	**0.04**
Height (cm)	168.4±10.9	169.8±11.1	168.9±11.7	167.3±10.6	0.865	0.260	0.477	0.245
BMI	35±8.7	36.7±10.1	33.6±6.9	35.3±9.2	0.057	0.522	0.295	0.065
Soft drink (%)	213 (64.5)	50 (68.5)	67 (62)	92 (66.2)	0.373	0.734	0.499	0.643
Chocolate (%)	259 (78)	51 (69.9)	84 (77.8)	115 (82.7)	0.232	0.033	0.330	0.098
Active smoking (%)	107 (32.4)	24 (33.3)	39 (36.1)	42 (30.2)	0.702	0.643	0.328	0.618
Alcohol (%)	107 (30)	22 (30.6)	37 (35.2)	47 (34.3)	0.517	0.584	0.880	0.798
Coronary artery disease (%)	100 (30.1)	16 (21.9)	43 (39.8)	39 (28.1)	**0.013**	0.334	0.053	**0.026**
Coronary artery bypass grafting (%)	32 (9.6)	8 (11)	13 (12)	11 (7.9)	0.824	0.463	0.281	0.537
Past myocardial infarction (%)	67 (20.2)	13 (17.8)	28 (25.9)	25 (18)	0.203	0.974	0.133	0.247
Congestive heart failure (%)	31 (9.3)	10 (13.7)	9 (8.3)	11 (7.9)	0.252	0.185	0.905	0.351
Diabetes mellitus (%)	132 (39.8)	26 (35.6)	42 (38.9)	59 (42.4)	0.656	0.336	0.573	0.614
Hypertension (%)	263 (79.2)	57 (78.1)	84 (77.8)	113 (81.3)	0.961	0.577	0.495	0.757
Atrial fibrillation/flutter (%)	42 (12.7)	11 (15.1)	11 (10.2)	19 (13.7)	0.327	0.781	0.407	0.579
Chronic obstructive pulmonary disease (%)	73 (22)	15 (20.5)	24 (22.2)	33 (23.7)	0.788	0.598	0.779	0.866
Asthma (%)	72 (21.7)	11 (15.1)	27 (25)	28 (20.1)	0.111	0.366	0.364	0.265
Stroke (%)	36 (10.8)	10 (13.7)	9 (8.3)	16 (11.5)	0.252	0.645	0.413	0.504
Chronic kidney disease (%)	11 (3.3)	4 (5.5)	3 (2.8)	3 (2.2)	0.364	0.214	0.754	0.405
Gastroesophageal reflux disease (%)	103 (31)	30 (41.1)	15 (13.9)	56 (40.3)	**<0.001**	**0.909**	**<0.001**	**<0.001**
Hyperlipidemia (%)	229 (69)	53 (72.6)	80 (74.1)	90 (64.7)	0.826	0.247	0.118	0.237
Peripheral vascular disease (%)	38 (11.4)	8 (11)	10 (9.3)	17 (12.2)	0.708	0.785	0.459	0.759
Antiplatelet (%)	163 (49.1)	27 (37)	66 (61.1)	65 (46.8)	**0.002**	0.173	**0.026**	**0.004**
Beta blockers (%)	155 (46.7)	32 (43.8)	54 (50)	66 (47.5)	0.416	0.256	0.695	0.718
Calcium channel blockers (%)	88 (26.5)	16 (22.2)	34 (31.5)	(25.9)	0.176	0.557	0.335	0.364
Statins (%)	159 (47.9)	33 (45.2)	55 (50.9)	66 (47.5)	0.450	0.752	0.591	0.737
Proton-pump inhibitors (%)	102 (30.7)	23 (31.9)	32 (29.6)	46 (33.1)	0.741	0.866	0.561	0.843
Diuretics (%)	136 (41)	27 (37.5)	37 (34.3)	67 (48.2)	0.656	0.139	**0.028**	0.068
ACEI-ARB (%)	152 (45.8)	32 (44.4)	43 (39.8)	71 (51.1)	0.537	0.361	0.079	0.205
H2-Blockers (%)	24 (7.2)	5 (6.9)	6 (5.6)	11 (8)	0.704	0.071	0.461	0.760

Angiotensin-converting enzyme inhibitor. ARB: Angiotensin receptor blocker

^1^ Comparison between non-coffee drinker (group 1) and subject who drank coffee 12–24 hours prior (group 2)

^2^ Comparison between non-coffee drinker (group 1) and subjects who drank coffee more than 24 hours prior (group 3)

^3^ Comparison between subjects who drank coffee 12–24 hours (group 2) and subjects who drank coffee more than 24 hours prior (group 3)

^4^ Comparison between group 1, 2 and 3

SBP change (15.2±17.1 vs. 7.2±10.2 mmHg, p = 0.001), HR change (32.2±14 vs. 27.3±9.6 bpm, p = 0.038) and %MPHR (65.5±15.6 vs. 60.7±8.6%, p = 0.038) were significantly higher in non-coffee drinkers (group 1) compared to those who drank coffee 12–24 hours prior (group 2). %Change HR (44.8±19.7 vs. 40.7±15.76%, p = 0.377) was not significantly different between group 1 and 2.

**Fig 1:**
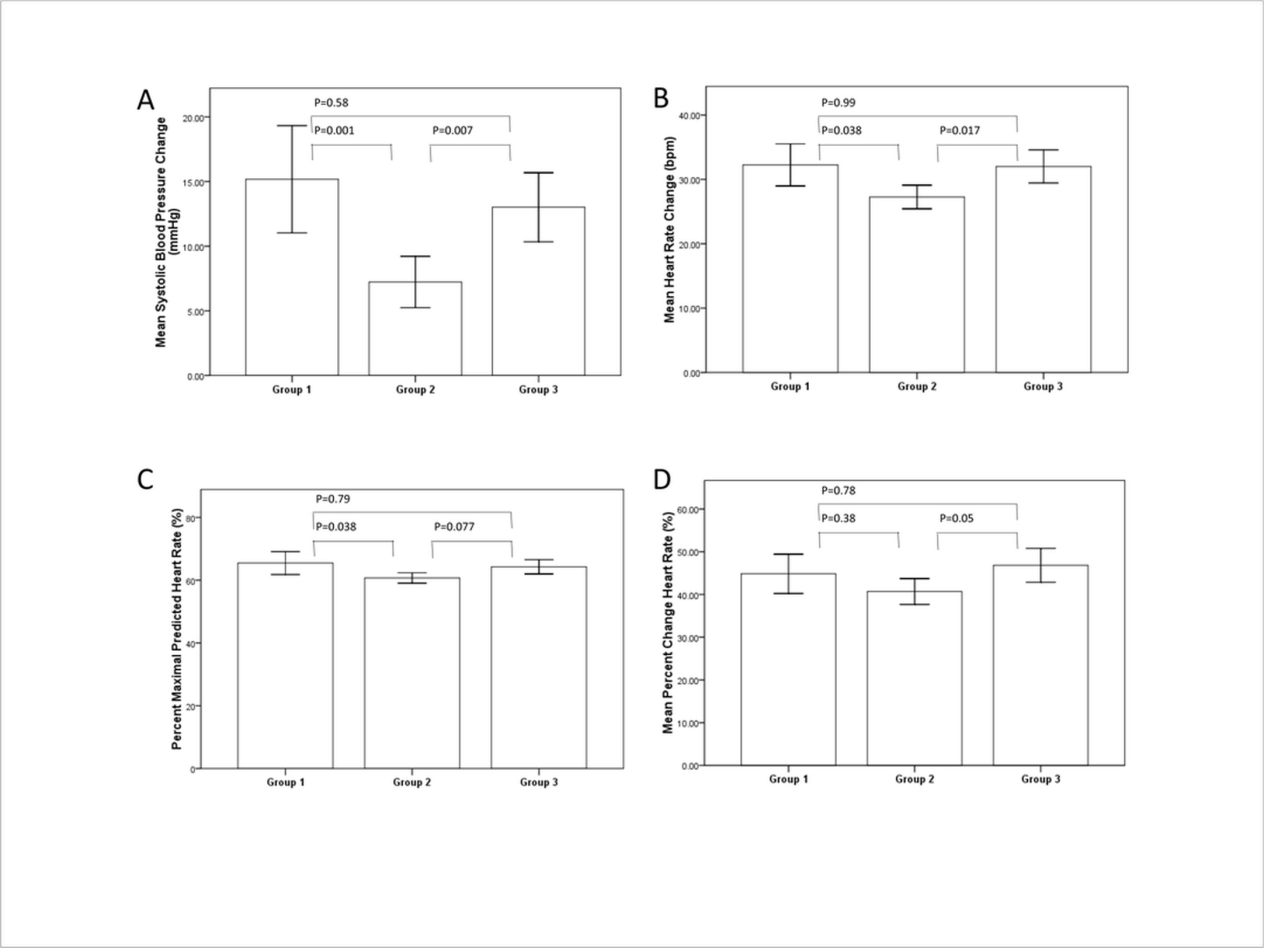
Regadenoson Effect on SBP change, HR change, %MPHR and % changeHR According to Coffee Consumption. Group 1: non-coffee drinkers; Group 2: subjects who drank coffee 12–24 hours prior to stress test; Group 3: subjects who drank coffee more than 24 hours prior to stress test. Error bars correspond to 95% confidence interval.

There was no significant difference in SBP change (15.2±17.1 vs. 13.01±15.8 mmHg, p = NS), HR change (32.2±14 vs. 32.1±15.3 bpm, p = NS), %MPHR (65.5±15.6 vs. 64.3±13.6%, p = NS), and %Change HR (44.8±19.7 vs. 46.8±23.7%, p = NS) between non-coffee drinkers (group 1) and those who drank coffee >24 hours prior (group 3). Moreover, subjects who drank coffee >24 hours prior (group 3) exhibited higher SBP change (13±15.8 vs. 7±10.2, p = 0.007) and HR change (32.1±15.3 vs. 27.3±9.6, p = 0.017) as compared to those who drank coffee 12–24 hours prior to testing (group 2). MPHR (64.3±13.6 vs. 60.7±8.6%, p = 0.077) and %Change HR (46.82±23.7 vs. 40.7±15.76%, p = 0.053) were higher among group 3 compared to group 2 but failed to achieve statistical significance (Table 2) (Fig 1).

**Table 2:** Hemodynamic Effects of Regadenoson According to Coffee Consumption.

	Non Coffee drinkers	12–24 hours hold	>24 hours hold	P Value^1^	P Value^2^	P Value^3^	P Value^4^
Baseline HR (bpm)	73.3±12.0	69.3±11.9	71.4±14.3	0.107	0.587	0.402	0.121
Peak HR (bpm)	105.5±19.5	96.5±14.1	103.4±20.5	**0.004**	**0.709**	**0.010**	**0.002**
HR change (bpm)	32.3±14.0	27.3±9.6	32.1±15.3	**0.038**	0.992	**0.017**	**0.010**
Baseline SBP (mmHg)	131.6±20.9	139±22.8	135.3±21.0	0.070	0.470	0.388	0.084
Baseline DBP (mmHg)	82.1±16.7	78.1±11.2	79.3±12.6	0.287	0.623	0.734	0.143
Peak SBP (mmHg)	145.2±24.6	145.5±21.9	151.7±46.6	0.994	0.791	0.667	0.289
Peak DBP (mmHg)	82±13.9	76.9±12.4	80.9±12.3	**0.024**	0.820	**0.041**	**0.014**
Change in SBP (mmHg)	15.2±17.1	7.2±10.2	13.0±15.8	**0.001**	0.574	**0.007**	**0.001**
Change in DBP (mmHg)	0.96±12.5	-0.97±6.6	1.5±12.1	0.482	0.946	0.191	0.206
% MPHR	65.5±15.7	60.7±8.6	64.3±13.6	**0.038**	0.791	0.077	**0.026**
%Change HR	44.8±19.7	40.7±15.8	46.8±23.7	0.38	0.779	0.053	0.065

HR: heart rate. SBP: systolic blood pressure. DBP: diastolic blood pressure. MPHR: maximal predicted heart rate. % ChangeHR: percent change in heart rate

^1^ Comparison between non-coffee drinker (group 1) and subject who drank coffee 12–24 hours prior (group 2)

^2^ Comparison between non-coffee drinker (group 1) and subjects who drank coffee more than 24 hours prior (group 3)

^3^ Comparison between subjects who drank coffee 12–24 hours (group 2) and subjects who drank coffee more than 24 hours prior (group 3)

^4^ Comparison between group 1, 2 and 3

After adjusting for age, race, weight, chocolate consumption, diuretics use, history of coronary artery disease, past myocardial infarction, asthma, calcium channel blocker and beta blocker use, Change SBP (p = 0.003), Change in HR (p = 0.046) and %MPHR (p = 0.015) remained significantly different between non-coffee drinkers (group 1) and subjects who consumed coffee 12–24 hours prior (group 2) in multivariable regression analysis. Moreover, on multivariable regression analysis, Change SBP (p = 0.031), Change HR (p = 0.019), and %Change HR (p = 0.041) remained significantly different between subjects who drank coffee 12–24 hours (group 2) and subjects who drank coffee more than 24 hours prior.

Among subjects who drank coffee 12–24 hours (group 2) prior to regadenoson administration, the number of coffee drinks did not have any effect on Change HR, Change SBP, MPHR and %Change HR (Table 3).

**Table 3:** Coffee Consumption Among Group With 12–24 Hour Hold.

Number of coffee cups consumed (n = 108)	1 (n = 5)	2 (n = 44)	3 (n = 36)	4 (n = 23)	P value^1^
HR_change (bpm)	27.3±3.3	27.5±8.8	27.4±9.5	26.7±11.9	0.990
SBP_change (mmHg)	5.5±6.8	6.7±10.5	9.0±11.9	5.6±6.8	0.603
% MPHR	59.7±5.3	60.2±7.7	61.7±9.5	60.3±9.2	0.858
%Change HR	40.0±11.9	42.9±16.3	39.7±17.7	40.7±15.7	0.630

HR: heart rate. SBP: systolic blood pressure. MPHR: maximal predicted heart rate. % ChangeHR: percent change in heart rate

^1^Comparison among group 2 (12–24 hour caffeine hold) based on number of coffee drink consumed

The number of self-reported adverse effects was lower in subjects exposed to caffeine 12–24 hours prior to regadenoson (group 2), as compared to >24 hours prior (group 3, or caffeine naïve subjects (group 1)(1.35±1.1 vs. 1.94±1.4 vs. 1.79±1.4; p = 0.002). Group 2 developed less abdominal pain (0.9% vs 16.4% vs. 14.4%, p<0.001), nausea (12% vs. 28.8% vs. 26.6%, p = 0.007) and dizziness (17.6% vs. 32.9% vs. 38.1%, p = 0.002) when compared to groups 1 and 3.

## Discussion

In our study, subjects who were coffee naive (group 1) or those who consumed coffee more than 24 hours prior (group 3) demonstrated significantly larger change in heart rate and systolic blood pressure when compared to subjects exposed to coffee within 12–24 hours (group 2) prior to regadenoson exposure.

The blunted rise in heart rate and systolic blood pressure observed in subjects with recent exposure to caffeine may be attributed to the long caffeine half-life in some patients. Caffeine (1,3,7-trimethylxanthine) bioavailability is 100%, with peak level achieved within 15 to 45 minutes [12]. It is metabolized predominantly by cytochrome P450 (CYP1A2 isoenzyme) with a half live (t1/2) ranging between 2 to 12 hours [12,13]. Many conditions and medications have been reported to affect caffeine metabolism and potentially affect its half-life. CYP450 inhibitors such as cimetidine, oral contraceptive usage, pregnancy, and alcoholic liver disease can increase caffeine half-life [14–16]. CYP450 inducers, such as phenytoin, phenobarbital, or rifampin, as well as smoking have been associated with a shortened caffeine half-life [17–18]. Caffeine is metabolized into 3 active metabolites: paraxanthine (1,7-dimethylxanthine), theobromine (3,7-dimethylxanthine) and theophylline (1,3-dimethylxatnhine)[19]. In humans, these metabolites account for 84%, 12% and 12% of caffeine metabolism respectively [20]. The half-life of paraxanthine, theobromine and theophylline can be as high as 4, 7 and 6 hours respectively, and these active metabolites can therefore extend the biologic effects of caffeine exposure [21].

Caffeine is a competitive inhibitor of adenosine A1, A2A and A2B receptors [22]. Chronic inhibition of adenosine receptors by caffeine results in increased sensitivity and up-regulation of those receptors [23,24]. Selective A2A receptors agonists, like regadenoson, are believed to increase heart rate by a reflex increase in sympathetic activity triggered by their vasodilatory effect on peripheral A2A receptors [25]. However, more recent evidence indicates a chemoreceptor mediated activation of the sympathetic nervous system and release of catecholamines [26,27]. In the coronary circulation, there is a high reserve for A2A receptor mediated coronary vasodilation with 25% receptor occupancy by regadenoson translating into 90% maximal vasodilation [10,28]. Thus minimal competitive inhibition by caffeine has been thought not to significantly impact maximal myocardial blood flow. In contrast, in the peripheral vasculature, A2A receptor reserve may be lower, thus possibly explaining a blunted increase in heart rate in response to regadenoson in subjects exposed to caffeine [10]

In addition, A2A receptors are present in the atrium and are able to activate ryanodine receptors [29]. Ryanodine receptor activation and subsequent calcium release mediates beta adrenergic heart rate stimulation [30]. A2A receptor agonists have been shown to modulate the response to beta adrenergic stimulation by attenuating the effect of A1 receptor and increasing contractility directly [31]. Therefore it is possible that caffeine exposure and partial inhibition of A2A receptors leads to decreased ryanodine receptor activation and blunting of beta adrenergic response mediated by A1 receptor. Our study findings suggest that consumption of caffeine within 24 hours may inhibit the vasodilatory properties of regadenoson administered for MPI. Moreover altered heart rate and blood pressure response could have been in response to a combination of altered sympathetic tone, variable chemoreceptor response, and vasodilatory effects by caffeine and regadenoson. We observed a decreased incidence of patient reported side effects in subjects exposed to caffeine between 12–24 hours supporting the possibility that residual caffeine activity attenuates the biologic response to regadenoson within this time window. It has been previously demonstrated that caffeine administered in doses of 200 or 400mg 90 minutes before regadenoson significantly reduces the sensitivity of MPI [11]. Following caffeine administration the mean number of reversible defects identified was reduced by approximately 60% [11]. It is therefore possible that based on variations in caffeine metabolism, exposure to caffeine between 12–24 hours before regadenoson could also reduce the sensitivity of regadenoson MPI. It may be necessary to hold caffeine consumption for 24 hours prior to regadenoson MPI to avoid any residual interaction.

Our study had many limitations. First, we did not measure blood caffeine level and thus we did not assess the association between serum caffeine level and effect on HR, BP and %MPHR response. Second, patient recall bias of caffeine consumption might have contributed to our results. We could not control for the potential possibility of patients underreporting their caffeine consumption in order to avoid stress test delay or cancelation. Altered sympathetic tone, variable chemoreceptor response, as well as vasodilatory effects by caffeine and regadenoson could have contributed to changes in heart rate and blood pressure response. Moreover, it is difficult to assess variations in caffeine dosing based on beverage types.

## Conclusions

Caffeine exposure within 12–24 hours prior to regadenoson MPI seems to attenuate the hemodynamic effect of regadenoson as indicated by blunting of heart rate and systolic blood pressure rise. Further studies are needed to examine the possible impact of caffeine exposure within 12–24 hours as currently endorsed in the FDA label on diagnostic sensitivity and specificity of regadenoson stress imaging.
